# ZFP36 Facilitates Senecavirus A (SVA) replication by inhibiting the production of type I interferon

**DOI:** 10.1016/j.virusres.2024.199498

**Published:** 2024-11-18

**Authors:** Mengge Yin, Lingyu Guan, Min Zhang, Xiangmin Li, Ping Qian

**Affiliations:** a National Key Laboratory of Agricultural Microbiology, Hubei Hongshan Laboratory, Huazhong Agricultural University, Wuhan, Hubei 430070, China; b College of Veterinary Medicine, Huazhong Agricultural University, Wuhan, Hubei 430070, China; c Key Laboratory of Preventive Veterinary Medicine in Hubei Province, The Cooperative Innovation Center for Sustainable Pig Production, Wuhan, Hubei 430070, China; d Key Laboratory of Development of Veterinary Diagnostic Products, Ministry of Agriculture of the People's Republic of China, Wuhan, Hubei 430070, China

**Keywords:** Zinc finger protein 36, Senecavirus A, ZF domain, VP1, MAVS

## Abstract

Zinc finger proteins (ZFPs) play an important role in the host-virus interplay. Zinc finger protein 36 is a member of the zinc finger protein 36 family, which includes two other paralogs, namely ZFP36L1 and ZFP36L2. Studies have demonstrated that ZFP36L1 acts as a host defender against influenza A virus and flaviviruses. However, the role of ZFP36 in host-virus interactions has not been thoroughly investigated. Here, we demonstrated that human zinc finger protein 36 (hZFP36) exhibited potent pro-viral activity during Senecavirus A infection. Overexpression of ZFP36 facilitated Senecavirus A infection, while hZFP36 knockdown inhibited viral replication. The ZF motifs of hZFP36 are key for promoting viral proliferation. hZFP36 stabilized Senecavirus A VP1 by binding to it. Furthermore, hZFP36 inhibited SeV-mediated IFN-β production through inducing caspase-dependent cleavage for MAVS. These findings provide insights into the mechanism of action of ZFP36 in host-virus interactions.

## Highlights

• Human zinc finger protein 36 (hZFP36) enhances Senecavirus A (SVA) replication.
• The ZF motifs of hZFP36 are key for its proviral activity.
• hZFP36 inhibits IFN-β production to promote SVA replication.
• hZFP36 induces caspase-dependent cleavage for MAVS to inhibit IFN-β production.

## 1 Introduction

Senecavirus A (SVA), also known as Seneca Valley Virus (SVV), is a member of the *Picornaviridae* family (Hales et al., 2008). Researchers first discovered SVA in contaminants of the PER C6 cell line, and further studies showed that it is an oncolytic virus that selectively infects and kills human tumour cells (Hales et al., 2008; Reddy et al., 2007). SVA is a nonenveloped positive single-stranded RNA virus, and its viral genome consists of three components (ORF, 5′ UTR, and 3′UTR). The ORF-encoded polyprotein is cleaved into four structural (VP4, VP2, VP3, and VP1) and eight non-structural proteins (L, 2A, 2B, 2C, 3A, 3B, 3C, and 3D) (Hales et al., 2008; Meng et al., 2022). Structural proteins primarily participate in the invasion and assembly of virions. Specifically, VP1, VP2, and VP3 are involved in the formation of the viral capsid and serve as the main protective antigens. The capsid proteins exhibit multiple neutralizing epitopes, and stimulate hosts generate neutralizing antibodies. Moreover, structural proteins are essential for binding to receptors on the cell surface, when SVA virions entry into target cells (Gimenez-Lirola et al., 2016; Liu et al., 2020; Wang et al., 2023). SVA infection was later found to be associated with idiopathic vesicular disease in pigs, which has clinical symptoms very similar to vesicular diseases, such as foot-and-mouth disease (FMD) (Buckley et al., 2019; Xu et al., 2017). During infection, the affected animals range from initial anorexia, lethargy and fever to vesicular lesions on the mouth and hoof. In addition, the survival rate of neonatal piglets is reduced when SVA infects piglets (Joshi et al., 2016; Maggioli et al., 2018; Wu et al., 2022). As an emerging infectious disease pathogen with global epidemic potential, there are currently no commercial vaccines and no specific drugs (Liu et al., 2020; Pasma et al., 2008; Saeng-Chuto et al., 2018; Sun et al., 2017; Vannucci et al., 2015; Xu et al., 2017). Therefore, a better understanding of host-virus interplay can help to identify potential targets for the development of potent antiviral drugs and vaccines.

Zinc finger proteins (ZFPs) are implicated in diverse cellular processes, and are involved in the host-virus interplay (Cassandri et al., 2017; Jen and Wang, 2016; Singh and van Attikum, 2021; Wang and Zheng, 2021). According to the arrangement of cysteines and histidines within the zinc ion-binding finger-like (ZnF) motif, ZFPs are categorized in subfamilies C4, C6, C8, C2 HC, C2 HC5, C2 H2, C3 HC4, CCCH, and C4HC3 (Cassandri et al., 2017). CCCH-type ZFPs (also termed RNA-binding proteins) are one of the most common subfamilies of eukaryotes with one or more CCCH domains whose functions include RNA metabolism, ubiquitination, and transcriptional repression (Hajikhezri et al., 2020; Hall, 2005; Klug, 2010; Maeda and Akira, 2017). CCCH-type zinc finger protein 36 (ZFP36), also known as TTP, TIS11, G0S24, and NUP475, is best-studied humans CCCH-type ZFPs and a member of the zinc finger protein 36 family, which includes two other paralogs, namely ZFP36L1 and ZFP36L2 (Fu and Blackshear, 2017). ZFP36 is involved in regulating the expression of various host genes by affecting the stability and translation of mRNA. ZFP36 can specifically recognize and bind to AU-rich region of the 3′-untranslated region of the target mRNA, including the ATG16L1 mRNA, TNFα mRNA, IL6 (interleukin 6) mRNA, IL8 mRNA, and so on (Fu and Blackshear, 2017; Lai et al., 2000; Stockwell et al., 2017; Zhang et al., 2020). Most of the proteins in the zinc finger protein family are RNA-binding proteins. Studies have shown that zinc finger proteins can affect the stability of target mRNAs by interacting with viral or host mRNAs. ZAP recognizes and binds to different families of single-stranded RNA viruses, thus exerting its antiviral activity (de Andrade and Cirne-Santos, 2023). During virus infection, ZNFX1 expression is upregulated and ZNFX1 bind to viral RNA, then induces MAVS-mediated antiviral innate immunity (Wang et al., 2019). ZNF281 inhibits mitochondrial biogenesis by directly binding to and regulating the TFAM promoter to suppress PGC-1α-NRF1-TFAM axis (Zhao et al., 2023).

Currently, studies of ZFP36 focus on the regulation of inflammatory factors and cytokine production. In this study, we screened SVA positive regulator hZFP36 and explored the molecular mechanism of hZFP36 promoting SVA proliferation. hZFP36 positively regulates SVA proliferation at an early stage of SVA proliferation. Furthermore, mutant hZFP36 (C124R/C162R) of hZFP36 does not affect SVA proliferation. In addition, overexpression of hZFP36 inhibits the production of type IFN though inducing caspase-dependent cleavage for MAVS, whereas the MAVS-D429A mutant can resist this cleavage. The above results provide new insights into subsequent research on SVA infection and antiviral drug design, and lay a foundation for in-depth understanding of the antiviral and immune regulatory functions of ZFP36.

## 2 Materials and methods

### 2.1 Cell culture, viruses, and reagents

HEK293T (Human embryonic kidney 293T) and BHK-21 (baby hamster kidney) cells were cultured in DMEM (Dulbecco's modified essential medium) containing 10% FBS (fetal bovine serum), 100 µg/mL streptomycin, and 100 U/ml penicillin at 37 °C.

SVA, pSKII-EGFP-SVA-HB, and Sev were stored in our laboratory. SVA and pSKII-EGFP-SVA-HB were propagated in BHK-21 cells, and the titers was determined though plaque assay.

The MG132, the z-VAD-FMK, DMSO, NH4Cl, protein A+G, and DAPI were obtained from Beyotime Biotechnology. Mouse anti-Tubulin monoclonal antibodies, mouse anti-ZFP36 monoclonal antibodies, rabbit anti-IRF3 polyclonal antibody, rabbit anti-Flag monoclonal antibody, mouse anti-GST monoclonal antibodies, rabbit anti-GST polyclonal antibody, mouse anti-HA monoclonal antibodies, rabbit anti-HA monoclonal antibody, and rabbit anti-PARP1 polyclonal antibody were obtained from Proteintech; Mouse anti-GAPDH monoclonal and rabbit anti-p-IRF3-386 monoclonal antibody were obtained from AB clonal; Mouse anti-Flag monoclonal was obtained from MBL; Rabbit anti-SVA VP1 polyclonal antibody was prepared in our laboratory.

### 2.2 Plasmids

All plasmids of SVA viral proteins, MDA5-Flag, RIG-Flag, TBK1-Flag, IKK-Flag, IRF3-Flag, and MAVS (WT and mutants)-Flag were stored in our laboratory. hZCCHC7 (GenBank accession GenBank accession number: NM_001289120.2), hTOE1 (GenBank accession number: NM_025077.4), hZC3H8 (GenBank accession number: NM_032494.3), hZC3H14 (GenBank accession number: NM_001160103.2), hZFP36 (GenBank accession number: NM_003407.5), and hZFP36L1 (GenBank accession number: NM_001244698.2) were amplified from HEK293T cells and were inserted into pTRIP-3Flag. Furthermore, the wild type and mutant plasmids of hZFP36 were cloned into pCDNA3.1-Flag and pCAGGS-HA.

### 2.3 RNA interference and transfection

Three siRNAs specifically targeting the hZFP36 mRNA were designed by Sangon Biotech (Shanghai). In this experiment, we used the siRNA sequences as follows: sihZFP36 (sense, 5′-CGCUACAAGACUGAGCUAUGUTT -3′; antisense, 5′-ACAUAGCUCAGUCUUGUAGCGTT-3′).

Overexpression and knockdown experiments were performed using JetPRIME (Polyplus Transfection, PT-114-15) according to the manufacturer's instructions.

### 2.4 Western blot, immunoprecipitation, and RNA immunoprecipitation

Western blot (WB) (Yin et al., 2022), Immunoprecipitation (IP) (Wen et al., 2022), and RNA immunoprecipitation (RIP) (Wen et al., 2022) were performed as previously described. Briefly, the cell samples are lysed with NP40 buffer (1.19% HEPES, 0.88% NaCl, 0.04% EDTA, 1% NP-40), and protein concentrations were determined using the BCA assay (Thermo Scientific). For IP, protein samples and the indicated antibody were incubated at 4 °C for 4 h, then protein A/G was added for another 4 h. Subsequently, the protein-antibody-beads mixture was washed with cold NP40. The protein was separated by SDS-PAGE and wet transferred to PVDF membranes. The membranes were blocked with 5% milk and incubated with appropriate primary antibody for indicated time, then the corresponding HRP-linked secondary antibodies were used to incubate for 1 h. For RIP, the washed RNA-protein-antibody-beads mixture added to TRIzol reagent. RT-PCR was performed to identify RNA-hZFP36 association.

### 2.5 Confocal microscopy

HEK293T cells were co-transfected with various plasmids for 24 h, and fixed in 4% (v/v) paraformaldehyde for 10 min at 4 °C, the cells blocked with 2% BSA for 2 h. The cells were then washed three times with PBS and incubated with appropriate primary antibody for indicated time. Then the cells were incubated with Alexa Fluor 488- or 555-conjugated antibodies for 1 h. DAPI (4’,6-diamidino-2-phenylindole) staining of nuclei for 10 min.

### 2.6 RNA extraction and quantitative RT-PCR

RNA Extraction and Quantitative RT-PCR were performed as previously described (Wen et al., 2022). In brief, cell samples were collected and total RNA was extracted, followed by reverse transcription to cDNA. Real-time qPCR was performed using Hieff UNICON Universal Blue qPCR SYBR green Master Mix following the manufacturer's instructions.

### 2.7 SVA binding and entry

HEK293T cells were transfected vector or hZFP36. After 24 h, the cells were pre-chilled at 4 °C for 1 h and infected with SVA (MOI=10) for 1 h at 4 °C. For binding assays, the cells were washed with pre-cold PBS and harvested for RT-qPCR and plaque assay. For entry assays, after SVA binding, the cells were incubated with 5% FBS at 37 °C for 1 h. The pre-cold alkaline high-salt solution (1 M NaCl and 50 mM sodium bicarbonate) was performed to wash the cells for at least three times. Subsequently, the cells washed with pre-cold PBS and collected for RT-qPCR and plaque assay.

### 2.8 Plaque assay

BHK-21 cells were seeded in 6-well plates and washed with PBS when the cells grew to 80%. Then, the cells were incubated with 0.8 mL ten-fold serial dilutions of virus. After 1.5 h, the cells were washed with PBS for one time and added 2 mL DMEM-LMP mixture containing 2% Low Melting-point agrose (LMP), 1*DMEM, 2% FBS and 1% Penicillin streptomycin solution. 10% (v/v) formaldehyde was used to fix cells for 2 h, then the cells were stained with 0.2% (m/v) crystal violet at least 15 min.

### 2.9 Luciferase reporter assay

HEK293T cells were co-transfected with luciferase plasmid, an internal control (pRL-TK) and target plasmid for 24 h. The dual-luciferase reporter assay system (Beyotime) tests the activity of firefly luciferase and Renilla luciferase.

### 2.10 Statistical analysis

GraphPad Prism software, version 8 was used to analyze all data. Comparisons between various treatments were done with an unpaired, two-tailed Student t test. Not statistically significant if *p > 0.05*.

## 3 Results

### 3.1 Screening of host factors affecting SVA proliferation

**Fig. 1:**
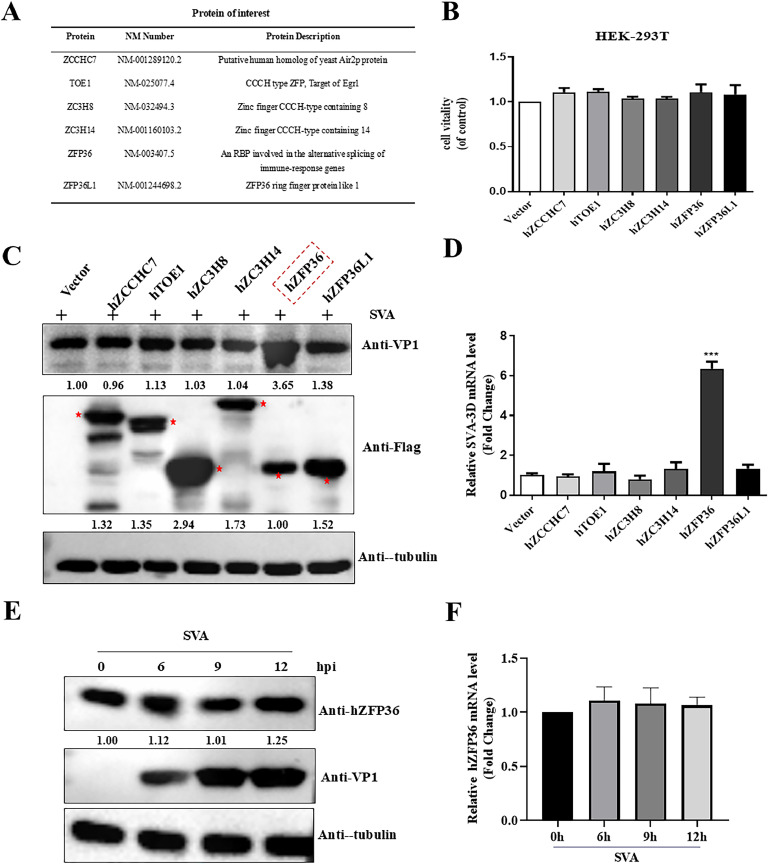
Screening of host factors affecting SVA proliferation. (A) Profile of a candidate zinc finger protein. (B) HEK293T cells were transfected with empty plasmids, or hZFP36, or hZFP36L1, or hZCCHC7, or hTOE1, or hZC3H8, or hZC3H14 for 24 h, and then the cells viability was measured by using Cell Counting Kit-8 (CCK8). (C and D) HEK293T cells were transfected with empty plasmids, or hZFP36, or hZFP36L1, or hZCCHC7, or hTOE1, or hZC3H8, or hZC3H14 for 24 h, and then the cells were infected with 0.1 MOI SVA for 8 h. The cells were collected and analyzed by Western blotting using the indicated antibodies, and the relative expression levels of SVA RNA were determined by RT-qPCR. (E and F) HEK293T cells were infected with 0.1 MOI SVA for the indicated times. The cells were collected for Western blotting and RT-qPCR. Data are shown as means ± SD. *, *P < 0.05*; **, *P < 0.01*; ***, *P < 0.001*.

Zinc finger proteins are involved in host-virus interactions and account for a large proportion of host antagonist viruses. It has attracted increasing attention because of its effect on most viral infections. SVA is an emerging infectious disease with a global epidemic for which there is no vaccine or specific drug to prevent and treat the disease. As shown in Fig. 1A, we selected six zinc finger proteins to test their effects on SVA proliferation. First, the six zinc finger protein genes were transfected into cells to detect their effects on cell viability. CCK-8 assay result showed that transfection of these six plasmids had no effect on HEK293T cell viability (Fig. 1B). Next, we next detected the effect of hZFP36, hZFP36L1, hZC3H14, hTOE1, hZC3H8, and hZCCHC7 on SVA proliferation. The result showed that the expression of SVA VP1 protein increased 3.65-fold (Fig. 1C) and SVA 3D gene mRNA increased 6.51-fold (Fig. 1D) due to overexpression of hZFP36, while the other five zinc finger proteins showed no significant effects on the proliferation of SVA. We also tested whether SVA had an effect on the expression of endogenous ZFP36. The Western blotting and RT-qPCR results indicated that SVA infection had no significant effect on the expression of hZFP36 protein and mRNA (Fig. 1 E and F).

### 3.2 hZFP36 promotes SVA replication

**Fig. 2:**
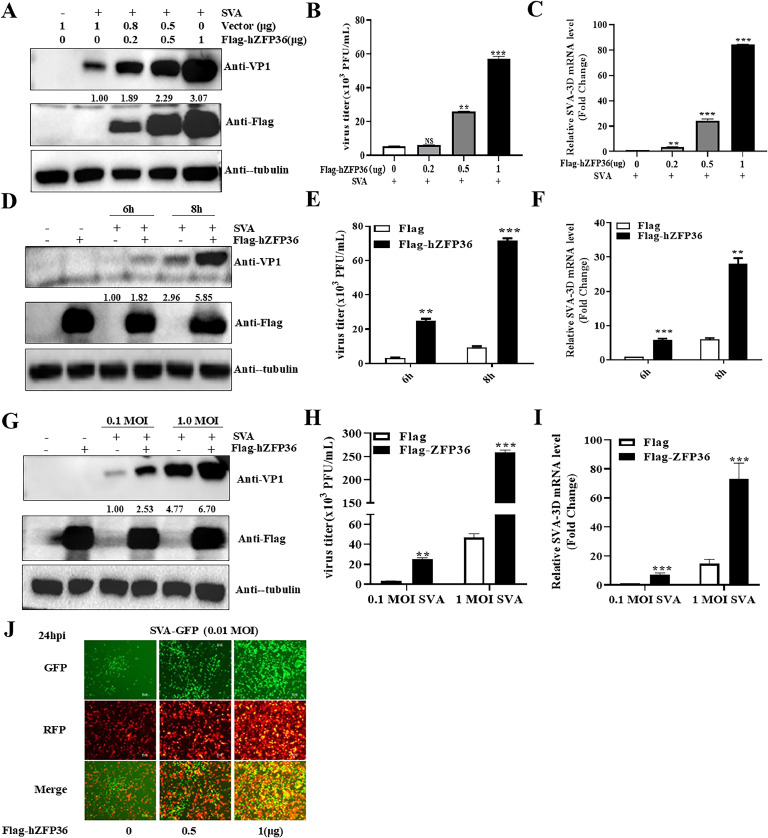
hZFP36 promotes SVA replication. (A–C) HEK293T cells were transfected with empty plasmids, or increased amounts of Flag-hZFP36 for 24 h, and then the cells were infected with 0.1 MOI SVA for 8 h. The cells were collected for Western blotting and RT-qPCR. The cells supernatant was collected to test SVA titers. (D–F) HEK293T cells were transfected with 1 μg empty plasmids, or Flag-hZFP36 for 24 h, and then the cells were infected with 0.1 MOI SVA for the indicated times. The cells were collected for Western blotting and RT-qPCR. The cells supernatant was collected to test SVA titers. (G–I) HEK293T cells were transfected with 1 μg empty plasmids, or Flag-hZFP36 for 24 h, and then the cells were infected with 0.1 or 1 MOI SVA for 8 h. The cells were collected for Western blotting and RT-qPCR. The cells supernatant was collected to test SVA titers. (J) HEK293T cells were transfected with empty plasmids, or increased amounts of pTRIP-3Flag-hZFP36 for 24 h. and then the cells were infected with 0.01 MOI pSKII-EGFP-SVA-HB for 24 h. Immunofluorescence microscopy observed the effect of hZFP36 on SVA infection. SVA is green, pTRIP-3Flag-hZFP36 (pTRIP-3Flag-hZFP36 expresses RFP-tagged hZFP36) is red and the nucleus is blue. Data are shown as means ± SD. *, *P < 0.05*; **, *P < 0.01*; ***, *P < 0.001.*

Based on the above finding, we further systematically evaluated the effect of hZFP36 on SVA proliferation. HEK293T cells were transfected with increased amounts of hZFP36 and then were infected with SVA. We observed that SVA VP1 protein expression (Fig. 2A), SVA mRNA level (Fig. 2C), and virus titers (Fig. 2B) significantly increased with increasing hZFP36. To assess the effect of ZFP36 on SVA infection at different time points, hZFP36-expressing cells were infected with 0.1 MOI SVA for 6 or 8 h. As shown in Fig. 2D and E, overexpressed hZFP36 resulted in increased VP1 expression and SVA titers. In cells overexpressing hZFP36, the expression of SVA VP1 protein increased 3.21-fold (Fig. 2D) and SVA titers increased 2.85-fold (Fig. 2E) at 8 h post infection compared to 6 h post infection (hpi). Consistent with Western blotting and plaque assay results, RT-qPCR results showed that the levels of SVA mRNA significantly increased in the hZFP36 group (Fig. 2F). Meanwhile, hZFP36-expressing cells were infected with different doses of SVA. The Western blotting, plaque assay, and RT-qPCR results showed that hZFP36 promoted SVA infection at different MOIs (0.1 MOI or 1 MOI) (Fig. 2G–I). In addition, HEK293T cells were transfected with empty plasmids, or increased amounts of hZFP36 for 24 h, and then the cells were infected with recombinant GFP-expressing SVA (SVA-GFP) for another 24 h. Along with increasing hZFP36, the GFP fluorescence signal significantly increased (Fig. 2J), indicating that hZFP36 promotes SVA replication. Taken together, these results suggest that hZFP36 facilitates SVA replication.

### 3.3 hZFP36 knockdown inhibits SVA replication

**Fig. 3:**
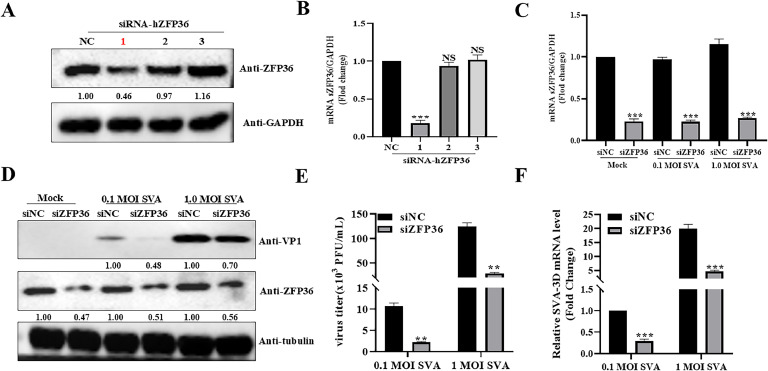
hZFP36 knockdown inhibits SVA replication. (A and B) HEK293T cells were transfected with siNC or siZFP36 for 24 h, and then the cells were collected for Western blotting and RT-qPCR. (C–F) HEK293T cells were transfected with siNC or siZFP36 for 24 h, and then the cells were infected with 0.1 or 1.0 MOI SVA for 8 h. The cells were collected for Western blotting and RT-qPCR. The cells supernatant was collected to test SVA titers. Data are shown as means ± SD. *, *P < 0.05*; **, *P < 0.01*; ***, *P < 0.001*.

To further determine the effect of hZFP36 on SVA propagation, we knocked down endogenous hZFP36 using siRNA. The Western blotting and RT-qPCR results showed that sihZFP36-1 significantly reduced the endogenous expression level of hZFP36 (Fig. 3A and B). Next, HEK293T cells were transfected with negative control (siNC) or sihZFP36-1 for 24 h, and then the cells were infected with SVA. The RT-qPCR (Fig. 3C) and Western blotting (Fig. 3D) results indicated that hZFP36 was successfully knocked down in the sihZFP36 group. As shown in Fig. 3D–F, knockdown of hZFP36 dramatically decreased the expression levels of VP1, the mRNA levels of SVA 3D, and SVA titers. Thus, the hZFP36 protein is a positive regulatory factor of SVA replication.

### 3.4 hZFP36 promotes SVA replication early in the course of infection

**Fig. 4:**
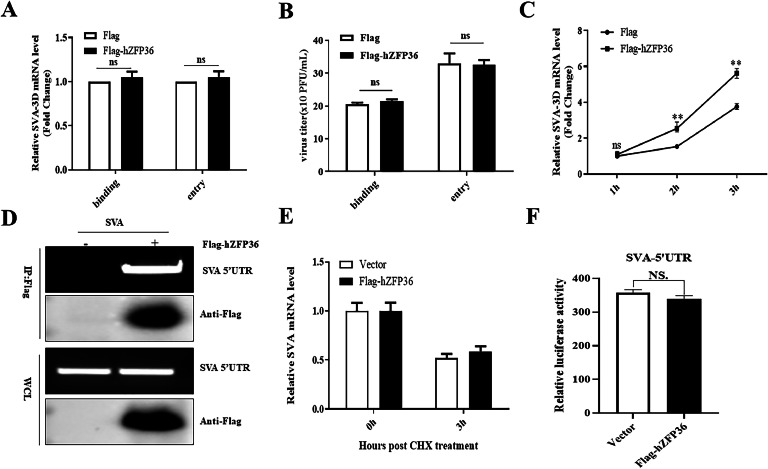
hZFP36 promotes SVA replication at the early stage of the life cycle. (A and B) HEK293T cells were transfected with 1 μg empty plasmids, or Flag-hZFP36 for 24 h. For binding assays, the cells were infected with 10 MOI SVA for 1 h at 4 °C. For entry assays, after incubation at 4 °C, the cells are incubated with 5% FBS at 37 °C for 1 h. The cells were collected for RT-qPCR. (C) HEK293T cells were transfected with 1 μg empty plasmids, or Flag-hZFP36 for 24 h, and then the cells were infected with 1.0 MOI SVA for the indicated times. The cells were collected for RT-qPCR. (D) HEK293T cells were transfected with 1 μg empty plasmids, or Flag-hZFP36 for 24 h, and then the cells were infected with 1.0 MOI SVA for the indicated times. The cells were collected for RNA immunoprecipitation. (E) HEK293T cells were transfected with 1 μg empty plasmids, or Flag-hZFP36 for 24 h, and then the cells were infected with 1.0 MOI SVA at 4°C. After 1 h, the cells were washed five times with cold PBS and treated with cycloheximide (CHX) for indicated time points. The cells were collected for RT-qPCR. (F). HEK293T cells were transfected with 1 μg empty plasmids, or Flag-hZFP36, pRL-TK (as an internal control) and SVA-5’UTR-Luc for 24 h. Using the Dual-Luciferase Reporter Assay System Kit to measure SVA 5′UTR activity. Data are shown as means ± SD. *, *P < 0.05*; **, *P < 0.01*; ***, *P < 0.001*.

To investigate the role of hZFP36 at the stage of SVA replication, the viral binding and entry assay was performed in vector- or hZFP36-expressing cells. Vector- or hZFP36-expressing cells were supplemented with pre-chilled fresh medium and infected with SVA at 4 °C for 1 h. Subsequently, the cells were washed and supplemented with prewarmed fresh medium at 37 °C for 1 h. The RT-qPCR and plaque assay results suggested that hZFP36 did not block SVA binding or entry (Fig. 4A and B). Next, vector- or hZFP36-expressing cells were infected with SVA at indicated times. Our data indicated that overexpression of hZFP36 significantly promoted the level of SVA RNA at 2 and 3 hpi (Fig. 4C).

E Carballo 's research suggests that ZFP36 binds to RNA targets (Carballo et al., 1998). We performed the RIP experiment to detect whether hZFP36 interact with SVA RNA. As shown in Fig. 4D, hZFP36 could interact with SVA mRNA. However, the RNA stability experiment showed that hZFP36 did not affect the stability of SVA RNA (Fig. 4E). In addition, the SVA 5′UTR played a critical role in SVA translation and replication. To explore whether hZFP36 affected 5’UTR activity, empty plasmids, or hZFP36, pRL-TK and SVA-5’UTR-Luc were co-transfected into HEK-293T. Compared to vector group, overexpression of hZFP36 did not significant influence the activity of SVA 5’UTR (Fig. 4F). These results confirm that hZFP36 promotes SVA replication at the early stage of the life cycle.

### 3.5 hZFP36 promotes the proliferation of SVA through its ZF domain

**Fig. 5:**
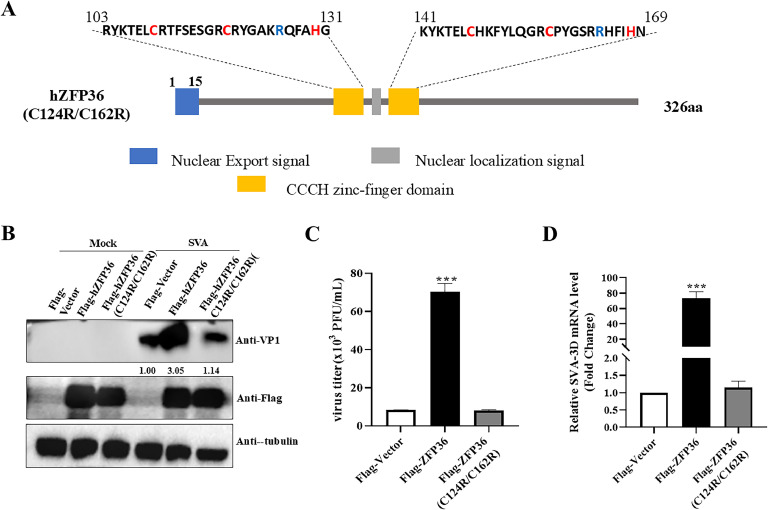
hZFP36 promotes the proliferation of SVA through its zinc-finger domain. (A) Diagram of the hZFP36 (C124R/C162R). (B–D) HEK293T cells were transfected with empty plasmids, or Flag-hZFP36 (WT and mutant) for 24 h, and then the cells were infected with 0.1 MOI SVA for the indicated times. The cells were collected for Western blotting and RT-qPCR. The cells supernatant was collected to test SVA titers. Data are shown as means ± SD. *, *P < 0.05*; **, *P < 0.01*; ***, *P < 0.001*.

The ZFP36 family consists of two tandem CCCH zinc finger motifs that are conserved and important for their biological functions (Chiu et al., 2022; Hodson et al., 2010; Lai et al., 2000; Morgan and Massi, 2010). We analyzed and constructed hZFP36 mutant (hZFP36 (C124R/C162R)) to assess its effect on SVA proliferation (Fig. 5A). HEK293T cells were transfected with empty plasmids, or hZFP36, or hZFP36 (C124R/C162R). After 24 h, the cells were infected with SVA for 8 h. Western blotting result showed that hZFP36 (C124R/C162R) mutant completely lost proviral activity on SVA proliferation (Fig. 5B). Simultaneously, the results of plaque (Fig. 5C) and RT-qPCR (Fig. 5D) assay results showed that overexpression of hZFP36 (C124R/C162R) showed no markedly effect on SVA proliferation, indicating that the ZF domains of ZFP36 are indispensable for its proviral activity.

### 3.6 hZFP36 interacts with VP1

**Fig. 6:**
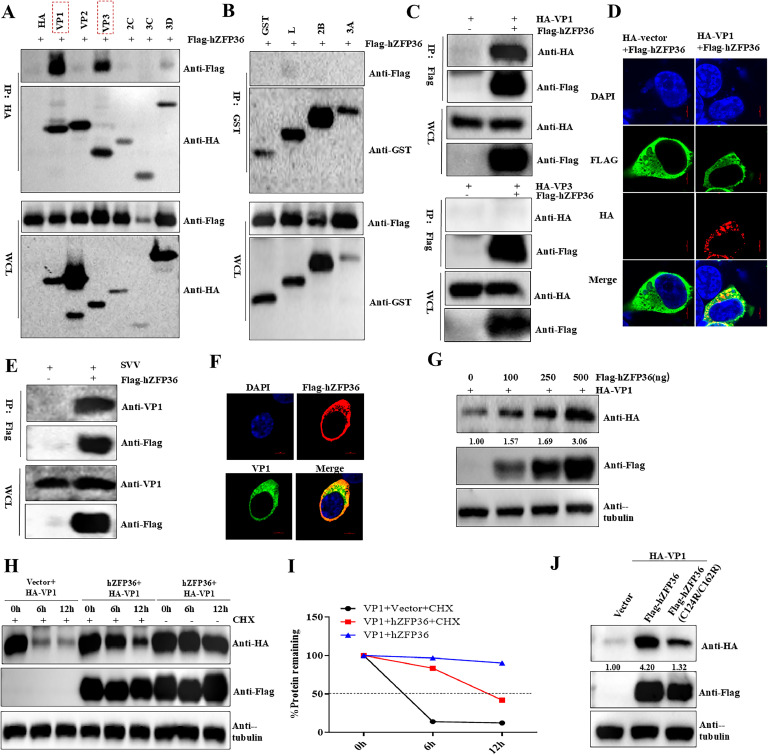
ZFP36 interacts with VP1. (A and B) HEK293T cells were co-transfected with 1 μg Flag-hZFP36 and viral proteins for 24 h, and then the cells were collected for Co-IP. (C) HEK293T cells were co-transfected with 1 μg HA-VP1 or HA-VP3 and 1 μg Flag-hZFP36 for 24 h, and then the cells were collected for Co-IP. (D) HEK293T cells were co-transfected with 0.2 μg HA-VP1 and 0.3 μg Flag-hZFP36 for 24 h, and the cells were analyzed by immunofluorescence staining with anti-Flag (green), anti-HA (red) and DAPI (blue) under confocal microscopy. (E) HEK293T cells were transfected with 1 μg Flag-hZFP36 for 24 h, and then the cells were infected with 1.0 MOI SVA. After 8 h, the cells were collected for Co-IP. **(F)** HEK293T cells were transfected with 0.5 μg Flag-hZFP36 for 24 h, and then the cells were infected with 1.0 MOI SVA. After 8 h, the cells were analyzed by immunofluorescence staining with anti-VP1 (green), anti-Flag (red) and DAPI (blue) under confocal microscopy. (G) HEK293T cells were co-transfected with increased amounts of Flag-hZFP36 and 0.5 μg HA-VP1 for 24 h, and then the cells were collected for Western blotting. (H and I) HEK293T cells were co-transfected with 0.5 μg Flag-hZFP36 and 0.5 μg HA-VP1, and the transfected cells were treated with CHX (100 µg/mL) for indicated times. Then the cells were collected for Western blotting. (J) HEK293T cells were co-transfected with 0.5 μg Flag-hZFP36 (WT and mutant) and 0.5 μg HA-VP1 for 24 h, and then the cells were collected for Western blotting.

Our data indicate that hZFP36 interacts with SVA mRNA but does not affect its stability (Fig. 4D and E). Previous research has also demonstrated that zinc finger proteins can interact with viral proteins, thereby influencing viral proliferation. To investigate the mechanism by which hZFP36 promotes SVA proliferation, Co-IP experiments were performed to test whether hZFP36 interacts with the SVA-encoded protein. As shown in Fig. 6A–C, hZFP36 did interact with SVA VP1, while other proteins did not interact with hZFP36. Simultaneously, immunofluorescence assay further supported that ectopic expression of hZFP36 and SVA VP1 colocalized in the cytoplasm (Fig. 6D). Furthermore, the interactions between VP1 and hZFP36 in SVA-infected cells was detected. The result showed that VP1 coprecipitated with the Flag-hZFP36 in SVA-infected cells (Fig. 6E). Besides, we also found that Flag-hZFP36 co-localized with SVA VP1 during authentic infection (Fig. 6F).

Because ZFP36 interacted with VP1, we examined its role in the protein expression level of VP1. Different doses of hZFP36 and VP1 were co-transfected into HEK-293T cells for 24 h, and the expression of SVA VP1 protein was detected by Western blot assay. The result showed that hZFP36 promoted the expression of the SVA VP1 protein in a dose-dependent manner (Fig. 6G). Next, the half-life of VP1 was investigated in CHX (cycloheximide)-treated HEK293T cells in the presence or absence of hZFP36. We observed that the overexpression of hZFP36 significantly extended the accumulation of VP1 (Fig. 6 H and I). In addition, we also investigated the effect of hZFP36 (C124R/C162R) on SVA VP1 by Western blot assay. As shown in Fig. 6 J, VP1 protein expression increased 1.32-fold in hZFP36 (C124R/C162R)-expressing cells; whereas VP1 protein expression increased 4.20-fold in hZFP36 (C124R/C162R)-expressing cells. The result indicated that VP1 protein expression decreased 3.18-fold in hZFP36 (C124R/C162R) group compared to the hZFP36 group. Altogether, the results indicate that hZFP36 interacts with VP1 and promotes VP1 expression by its ZF domain.

### 3.7 hZFP36 inhibits SeV-mediated IFN-β production

**Fig. 7:**
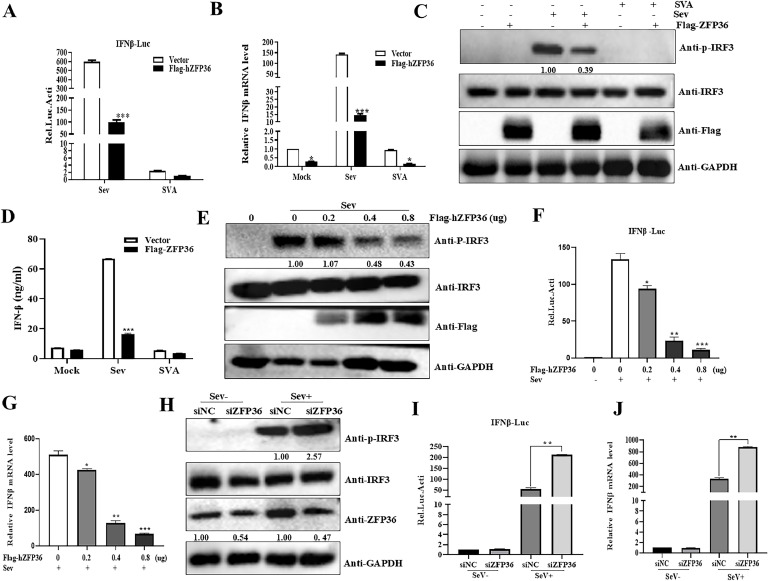
hZFP36 inhibits the production of IFN-β. (A) HEK293T cells were co-transfected with 0.5 μg HA-hZFP36 or pCAGGS-HA together with 100 ng IFN-β reporter and 5 ng pRL-TK. After 24 h, the cells were infected or uninfected with Sev (2^5^ HA) or SVA (MOI=0.01) for 12 h. Using the Dual-Luciferase Reporter Assay System Kit to measure IFN-β activity. (B–D**)** HEK293T cells were transfected 0.5 μg HA-hZFP36 or pCAGGS-HA for 24h, and then the cells were infected or uninfected with Sev (2^5^ HA) or SVA (MOI=0.01). After 12 h, cells were collected for RT-qPCR and immunoblot analysis. The supernatants were harvested to detect IFN-β secretion by ELISA assay. (E) HEK293T cells were co-transfected with increased amounts of HA-hZFP36 together with 100 ng IFN-β reporter and 5 ng pRL-TK. After 24 h, the cells were infected or uninfected with Sev (2^5^HA) or SVA (MOI=0.01) for 12 h. Using the Dual-Luciferase Reporter Assay System Kit to measure IFN-β activity. (F and G) HEK293T cells were transfected increased amounts of HA-hZFP36 for 24h, and then the cells were infected or uninfected with SeV (2^5^ HA) or SVA (MOI=0.01). After 12 h, cells were collected for RT-qPCR and immunoblot analysis. (H) HEK293T cells were transfected with siNC or siZFP36 for 12 h, and then the cells were co-transfected with 100 ng IFN-β reporter and 5 ng pRL-TK. After 24 h, the cells were infected or uninfected with Sev (2^5^HA) for 12 h. Using the Dual-Luciferase Reporter Assay System Kit to measure IFN-β activity. (I and J) HEK293T cells were transfected with siNC or siZFP36 for 36 h, and then the cells were infected or uninfected with Sev (2^5^ HA) for 12 h. The cells were collected for RT-qPCR and immunoblot analysis. Data are shown as means ± SD. *, *P < 0.05*; **, *P < 0.01*; ***, *P < 0.001*.

SVA is an IFN-β-sensitive virus (Qian et al., 2017). Comprehensive data demonstrated that most of zinc finger proteins influenced the IFN-I production. To explore whether hZFP36 is involved in the innate immune process during SVA infection, HEK293T cells were co-transfected with HA-hZFP36 and dual-luciferase reporter plasmids (IFN-β-Luc and pTK-Renilla). After 24 h, the cells were infected with Sev and SVA for 12 h, and the activity of IFN-β promoter was determined by luciferase assays. The result showed that hZFP36 inhibited the activation of the IFN-β promoter (Fig. 7A). The negative regulatory effect of hZFP36 on IFN production was also confirmed by detecting the mRNA level of IFN-β and the phosphorylation level of IRF3 (Fig. 7B and C). In addition, the ELISA result showed that hZFP36 suppressed the secretion of IFN-β protein (Fig. 7D). To further confirm that hZFP36 was a negative regulatory factor in the production of host type I interferon, increased amounts of hZFP36 were transfected into HEK293T cells to assess the activity of IFN-β promoter, the mRNA level of IFN-β and the phosphorylation level of IRF3. As shown in Fig. 7E, overexpression of hZFP36 inhibited Sev-induced activation of IFN-β promoter in a dose-dependent manner. With increasing hZFP36 concentration, the mRNA level of IFN-β was significantly inhibited (Fig. 7F). We also observed that Sev-induced phosphorylated IRF3 was reduced in a dose-dependent manner, when the cells were transfected with hZFP36 (Fig. 7G). To investigate the role of hZFP36 in Sev-mediated IFN-β production, the hZFP36 was knocked down by using siRNA. Downregulation of hZFP36 markedly facilitated Sev-triggered activation of IFN-β promoter (Fig. 7H), transcription of IFN-β genes (Fig. 7I) and phosphorylated IRF3 (Fig. 7J). Taken together, these results indicate that hZFP36 inhibits Sev-mediated IFN-β production.

### 3.8 hZFP36 induces caspase-dependent cleavage for MAVS to inhibit IFN-β production

**Fig. 8:**
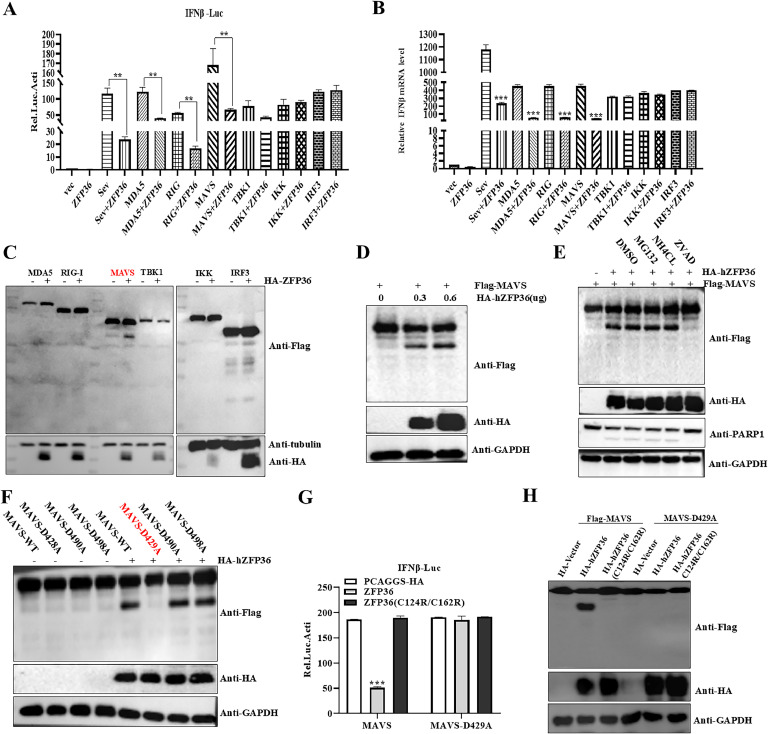
hZFP36 cleaved MAVS to inhibit IFN-β production. (A) HEK293T cells were 0.5 μg co-transfected various signaling components, 0.5 μg HA-hZFP36 together with 100 ng IFN-β reporter and 5 ng pRL-TK for 24 h. Using the Dual-Luciferase Reporter Assay System Kit to measure IFN-β activity. (B and C) HEK293T cells were transfected 0.5 μg various signaling components and 0.5 μg HA-hZFP36 for 24 h, and then cells were collected for RT-qPCR and immunoblot analysis. (D) HEK293T cells were co-transfected with increased amounts of HA-hZFP36 and 0.5 μg Flag-MAVS for 24 h, and then cells were collected for immunoblot analysis. (E) HEK293T cells were co-transfected with 0.6 μg HA-hZFP36 and 0.5 μg Flag-MAVS for 12h, and then cells were treated with MG132 (10 μM), NH4CL (10 mM), and z-VAD-FMK (50 μM) for 12 h. The cells were collected for immunoblot analysis. (F) HEK293T cells were co-transfected with 0.6 μg HA-hZFP36 and 0.5 μg Flag-MAVS (WT and mutant) for 24 h. The cells were collected for immunoblot analysis. (G) HEK293T cells were transfected with 0.6 μg HA-hZFP36 (WT or mutant), 0.5 μg Flag-MAVS together with 100 ng IFN-β reporter and 5 ng pRL-TK for 24 h. Using the Dual-Luciferase Reporter Assay System Kit to measure IFN-β activity. (H) HEK293T cells were transfected 0.6 μg HA-hZFP36 (WT or mutant) and 0.5 μg Flag-MAVS (WT and mutant). After 24 h, cells were collected for immunoblot analysis. Data are shown as means ± SD. *, *P < 0.05*; **, *P < 0.01*; ***, *P < 0.001*.

Given that hZFP36 inhibits Sev-mediated IFN-β production, HEK293T cells were co-transfected with various signaling components and hZFP36 to evaluate the role of hZFP36 on IFN-β production by these adaptor molecules. Luciferase assay result showed that hZFP36 inhibited MDA5-, RIG-I-, and MAVS-triggered IFN-β promoter activity (Fig. 8A). Similarly, MDA5-, RIG-I-, and MAVS-induced transcription of IFN-β was repressed (Fig. 8B). Furthermore, hZFP36 induced MAVS to produce an approximately 40 to 55 kDa fragment, but had no effect on the expression of MDA5 and RIG (Fig. 8C). To confirm the phenomenon, increased amounts of hZFP36 and MAVS were co-transfected into HEK293T cells. As shown in Fig. 8D, hZFP36 mediated this phenomenon in a dose-dependent manner. Next, we investigate the pathway hZFP36 induces MAVS fragment generation. HEK293T cells were co-transfected with hZFP36 and MAVS. After 12 h, MG132 (10 μM), NH4CL (10 mM), and z-VAD-FMK (50 μM) were applied for another 12 h. We found that hZFP36-mediated MAVS fragment generation hardly exists when z-VAD-FMK was used (Fig. 8E). Caspase-mediated cleavage can recognize the aspartic (D) residues of the target, we generated three mutants in which aspartic (D) residues were replaced by alanine (A) residues. Western blotting result showed that hZFP36 had no effect on MAVS-D429 mutant, indicating that hZFP36 induces caspase-dependent cleavage for MAVS at D429 (Fig. 8F). Overall, the results indicate that hZFP36 inhibit IFN-β production via mediating caspase-dependent cleavage for MAVS at a specific site. We further explored the function of hZFP36 (C124R/C162R) on MAVS- and MAVS-D429A-mediated signalling. As shown in Fig. 8G, hZFP36 significantly inhibited MAVS-mediated IFN-β promoter activity, but not hZFP36 (C124R/C162R). In addition, hZFP36 and hZFP36 (C124R/C162R) had no effect on MAVS-D429A-mediated IFN-β promoter activity. The Western blotting result also showed that hZFP36 (C124R/C162R) could not induce caspase-dependent cleavage for MAVS (Fig. 8H).

## 4 Discussion

Zinc finger proteins play an important role in the interaction between hosts and viruses (Vilas et al., 2018). Numerous studies have demonstrated that ZFP has both positive and negative effects on viral infections. Cellular Transcription Factor ZASC1 promotes Murine Leukemia Virus (MLV) gene expression by binding to the MLV 3′ LTR (Bruce et al., 2010). Zinc finger-containing CCCH type 11A (ZC3H11A) and the zinc finger-containing cellular transcriptional corepressor ZNF46 promote influenza A virus (IAV) replication through distinct mechanisms. ZC3H11A facilitates mRNA transport from the nucleus to the cytoplasm for multiple nuclear-replicating viruses (including adenovirus, HIV, herpes simplex virus, and influenza virus), while ZNF46 modulates the transcription activity of IAV RNA-dependent RNA polymerase (RdRp) to regulate its replication (Chen et al., 2017; Younis et al., 2018). Monocyte chemoattractant protein 1-induced protein 1 (MCPIP1) suppresses hepatitis C virus (HCV), Japanese encephalitis virus (JEV) and dengue virus (DENV) via its RNase activity and CCCH-type zinc finger domain (Lin et al., 2013; Lin et al., 2014). Additionally, Target of Egr1 (TOE1), Tristetraprolin (TTP) and zinc-finger antiviral protein (ZAP) exhibit different antiviral strategies against HIV-1 (Buckmaster and Goff, 2022; Maeda et al., 2006; Sperandio et al., 2015). This study aims to investigate the interaction between ZFP36 and SVA. Our results showed that SVA infection did not affect ZFP36. However, SVA replication was enhanced when ZFP36 was overexpressed in HEK29T cells. Furthermore, it was observed that the ZFP36 (C124R/C162R) mutant impaired its ability to promote SVA replication.

The SVA 5′ UTR contains an internal ribosome entry site (IRES), which is required for cap-independent translation initiation of SVA polyprotein and the replication of SVA (Hales et al., 2008; Hellen and de Breyne, 2007; Houston et al., 2020). Previous studies have shown that the ZFP36 family recognize and bind to RNAs to perform its functions. ZFP36L2 suppresses iTreg function by destabilizing and binding to the AU-rich elements of Ikzf2 mRNA (Makita et al., 2020). The pro-cancer gene HIF1A contains ARE motifs in its 3′UTR, which is critical for ZFP36L1 to directly interact with the 3ʹUTR of HIF1A mRNA and mediate HIF1A mRNA destabilization (Loh et al., 2020). Loss of ZFP36 function results in the proliferation, migration and invasion of NSCLC cells. Mechanistically, BARX1 expression is associated with NSCLC, ZFP36 binds to 3’UTR of BARX1 mRNA to mediate its degradation (Zhang et al., 2023). In our study, RIP result showed that ZFP36 could bind to SVA 5’UTR, but had effect on the activity of SVA 5’UTR (Fig. 4D and F). Previous studies have shown that DHX30 bound SVV dsRNA and did not inhibit the activity of SVV 5’UTR, which may have an impact on virus infection (Wen et al., 2022). Similarly, our result indicated that ZFP36 may not affect 5′ UTR-mediated translation initiation, while played an important role in the early stages of SVA replication.

ZFP36 family has two conserved CCCH-type ZF domains that are pivotal for their biological functions (Morgan and Massi, 2010). ZF mutant C135R/C173R of ZFP36L1 lost its anti-JEV and DENV infectivity as well as JEV and DENV RNA binding activity (Chiu et al., 2022). Our study indicated that hZFP36 (C124R/C162R) mutant lost its function to promote SVA infection compared with wild-type hZFP36 (Fig. 5B–D). hZFP36 interacted with VP1 (Fig. 6A–D) and increased the protein expression of VP1 (Fig. 6E), but VP1 induction was prevented in HEK293T cells overexpressing hZFP36(C124R/C162R) mutant (Fig. 6F). These results strongly suggest that ZF domains are critical for ZFP36 to promote SVA proliferation. However, further studies of this interaction between hZFP36 and VP1 are required to determine the precise contribution of hZFP36 to SVA replication.

Antiviral innate immunity is a mechanism developed by the host to limit viral infection and protect the host (Akira et al., 2006). Studies have revealed that zinc finger proteins play key roles in innate immunity (Wang and Zheng, 2021). TRIM25 induces the antiviral activity of RIG-I by mediating the ubiquitination of the K63 linkage of RIG-I (Gack et al., 2007). IAV infection can stimulate the expression of RIG-I. Subsequently, overexpression of RIG-I induces the activation of IRF3, which ultimately leads to the production of type I IFN to inhibit IAV infection (Kandasamy et al., 2016; Ramírez-Martínez et al., 2013). Sun et al study shows that MCPIP1 inhibits RIG-I expression to attenuate the antiviral immune response and increase IAV replication at the later stage of the life cycle (Sun et al., 2018). ZFR is a negative regulator of type I interferon signal pathway. Knockdown ZFR result in IFNβ production, and a reduction in the replication of EMCV and CVB3 (Haque et al., 2018). We showed here that hZFP36 suppressed IFN-β promoter activity (Fig. 7A), whereas this phenomenon was absent in siRNA-mediated inhibition of endogenous hZFP36 (Fig. 7G). Interestingly, Jiang et al study shows that ZFP36 positively regulates type I interferon production by promoting K63-linked polyubiquitination of RIG-I (Jiang et al., 2023). However, our results suggested that hZFP36 inhibited RIG-I-mediated IFN-β promoter activity. We speculate that this may vary depending on where you are in relation to inter-lab variations. In addition, Jiang et al. study suggests that ZFP36 C118S/C162S has no effect on RIG-I-N-induced signaling, while our data indicate that hZFP36 (C124R/C162R) loses its ability to promote SVV infection and suppress MAVS-triggered IFN-β promoter activity.

KLF4 directly combine with the promoter of IFN-β gene and impair the binding of IFN-β promoter and IRF3 to inhibit type I interferon production (Luo et al., 2016). SVV 3C^pro^ negatively regulates type I interferon production by targeting MAVS, TANK, and TRIF for cleavage and RIG-I and IRF3 for degradation (Qian et al., 2017; Wen et al., 2019; Xue et al., 2018). Caspase-3 cleaves MAVS, and IRF3 to inhibit type I interferon production (Ning et al., 2019). In our studies, hZFP36 mediates caspase-dependent cleavage for MAVS to inhibit IFN-β production. Baou et al study has shown that overexpression of the ZFP36 family of proteins can cause apoptosis in a variety of cells (Baou et al., 2009). Our result also indicated that the cleavage fragments of MAVS disappeared when using the caspase inhibitor Z-VAD-FMK (Fig. 8E). Our previous research found that PSV 3C^pro^ cleaved MAVS dependent on cellular apoptosis, and the MAVS D429A mutation was resistant to PSV 3C^pro^-mediated cleavage. Furthermore, the cleavage fragments of MAVS had no effect on IFN-I production, and PSV 3C^pro^ could not inhibit MAVS D429A mutation-mediated IFN-I production (Yin et al., 2022). Consistent with this, hZFP36 mediates caspase-dependent cleavage for MAVS at D429 (Fig. 8F).

In conclusion, our work uncovers hZFP36 as a host factor that promotes SVA infection. The ZF domain of hZFP36 plays a key role in the induction of SVA infection and VP1 protein expression. Taken together, we have demonstrated for the first time that hZFP36 is a key host factor for SVA infection and provided a concise insight into the regulatory mechanism between ZFP36 and the virus.

## Funding

This work was supported by the National Key R & D Program of China (2022YFD1800800 and 2021YFD1800300) and the 10.13039/501100001809National Natural Science Foundation of China (32072841 and 32072901).

## CRediT authorship contribution statement

**Mengge Yin:** Writing – original draft. **Lingyu Guan:** Writing – original draft. **Min Zhang:** Writing – original draft. **Xiangmin Li:** Writing – review & editing. **Ping Qian:** Writing – review & editing.

## Declaration of competing interest

On behalf of my co-authors, I would like to submit the enclosed manuscript entitled “ZFP36 Facilitates Senecavirus A (SVA) Replication by Inhibiting the Production of Type I Interferon”, which we wish to be considered for publication in “Virus Research”. The work described has not been submitted elsewhere for publication, in whole or in part. All the authors listed have approved the manuscript and we declare no conflict of interest.

## Data Availability

Data will be made available on request.
